# First-pass myocardial perfusion assessment using eight-fold accelerated k-t BLAST stress DCE-MRI with rapid parametric mapping

**DOI:** 10.1186/1532-429X-11-S1-O34

**Published:** 2009-01-28

**Authors:** Aleksandra Radjenovic, Sven Plein, Neil Maredia, Sebastian Kozerke, John Biglands, John Greenwood, John Ridgway

**Affiliations:** 1 University of Leeds, Leeds, UK; 2 University and ETH, Zurich, Switzerland

**Keywords:** Myocardial Perfusion, Enhancement Ratio, False Positive Lesion, Median Percentage Change, Significant Coronary Stenos

## Background

First-pass myocardial perfusion assessment using dynamic contrast enhanced MRI (DCE-MRI) is still one of the most challenging CMR applications. Image quality, spatial and temporal resolution are limited by the need to acquire multiple slices as single shot acquisitions within a single heart beat, as the process of interest is transient and very rapid, especially under pharmacologically induced stress hyperaemia. This is why accelerated acquisition methods, such as k-t BLAST [1], could provide a significant improvement in the assessment of myocardial perfusion by CMR.

An optimised DCE-MRI sequence with eight-fold k-t BLAST acceleration was shown to provide a significant improvement in spatial resolution without loss of image quality[2], making these datasets very suitable for parametric mapping.

## Objective

To investigate the ability of eight-fold k-t BLAST accelerated stress perfusion DCE-MRI combined with a rapid parametric mapping algorithm to detect regions of ischaemia in a pilot cohort of patients with suspected coronary heart disease (CHD).

## Methods

The regional ethics review board's permission was obtained and ten patients (9 male) were recruited into this study (age range 46–69, mean 59). First-pass stress myocardial perfusion DCE-MRI was performed on a whole body 1.5 T MR scanner (Gyroscan Intera CV, Philips Medical Systems) with dedicated k-t BLAST acquisition and reconstruction software (GyroTools Ltd, Switzerland). An optimised DCE-MRI sequence (Table 1) allowed three uniformly prepared slices to be acquired in every heart beat, for heart rates of up to 100 bpm [2]. DCE-MRI was acquired under adenosine induced stress with peripheral venous administration of 0.1 mmol/kg Gd-DTPA. The algorithm for quantitative analysis and parametric mapping comprised the following steps: 1) automated detection of the target post-contrast frame 2) endocardial border detection in the target post-contrast frame using automated region-growing algorithm and a single spline fitting to define epicardial border 3) automated registration of pre- and post-contrast frames using incremental rigid translation 4) computation of percentage enhancement ratios (ER) on voxel-by-voxel basis 5) histogram analysis of the ER datasets and creation of percentile-based colour maps.

**Table 1:** DCE-MRI sequence parameters

Saturation recovery pre-pulse delay	150 ms
TFE readout	TR/TE/φ = 3.6/1.7/15°
k-t acceleration factor	8
Image matrix	192 × 187

## Results

**Figure 1:**
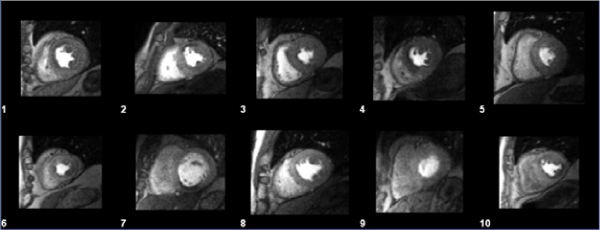
**Representative dynamic frames from ten accelerated DCE-MRI datasets**.

**Figure 2:**
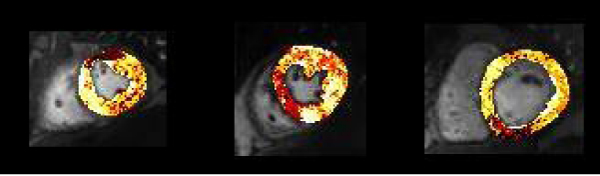
**Examples of parametric maps of enhancement ratios in single-vessel disease (corresponding to images 2, 4 and 7 in Figure 1)**. Dark coloured voxels belong to the lowest part of the individual study's frequency distribution.

On coronary X-ray angiography, 8/10 patients had coronary stenosis ≥ 70%, while two had no significant lesions (Table 2). Representative dynamic frames from all ten DCE-MRI studies are shown in Figure 1. Example parametric maps of ER are presented in Figure 2. Global enhancement ratios were computed as a median percentage change over baseline (Table 2).

**Table 2:** Median Enhancement Ratios (ER%)

Patient	1	2	3	4	5	6	7	8	9	10
Coronary artery X-ray (number of affected territories)	1	1	1	1	0	2	1	0	1	3
Median Enhancement Ratio%	61.4	133	64.6	38.3	77.2	33.1	58.0	65.2	40.6	19.1

Motion correction was not required in three datasets, and 1–2 voxel displacement was applied in seven datasets. In patients with no significant coronary stenoses, the average ratio was 71.2% (n = 2), in single-vessel disease it was 66.0% (n = 4). In a patient with two-vessel disease ER was 33.1% and in a patient with three-vessel disease ER was 19.1%.

## Discussion

The results of this pilot study suggest that the proposed methods for acquisition and analysis of first-pass myocardial perfusion are robust and ready for use in clinical studies, where its diagnostic utility needs to be assessed formally in a larger patient cohort. The methods allow the assessment of regional differences in perfusion, as well as global changes in perfusion. There is scope for further improvement, notably in increasing resistance to motion artefact and reducing signal inhomogeneity, which can lead to the appearance of false positive lesions.
